# Stereospecificity control in aminoacyl-tRNA-synthetases: new evidence of d-amino acids activation and editing

**DOI:** 10.1093/nar/gkz756

**Published:** 2019-09-03

**Authors:** Mariia Yu Rybak, Alexey V Rayevsky, Olga I Gudzera, Michael A Tukalo

**Affiliations:** Department of Protein Synthesis Enzymology, Institute of Molecular Biology and Genetics of the NAS of Ukraine, 150 Zabolotnogo Street, 03143, Kyiv, Ukraine

## Abstract

The homochirality of amino acids is vital for the functioning of the translation apparatus. l-Amino acids predominate in proteins and d-amino acids usually represent diverse regulatory functional physiological roles in both pro- and eukaryotes. Aminoacyl-tRNA-synthetases (aaRSs) ensure activation of proteinogenic or nonproteinogenic amino acids and attach them to cognate or noncognate tRNAs. Although many editing mechanisms by aaRSs have been described, data about the protective role of aaRSs in d-amino acids incorporation remained unknown. Tyrosyl- and alanyl-tRNA-synthetases were represented as distinct members of this enzyme family. To study the potential to bind and edit noncognate substrates, *Thermus thermophilus* alanyl-tRNA-synthetase (AlaRS) and tyrosyl-tRNA-synthetase were investigated in the context of d-amino acids recognition. Here, we showed that d-alanine was effectively activated by AlaRS and d-Ala-tRNA^Ala^, formed during the erroneous aminoacylation, was edited by AlaRS. On the other hand, it turned out that d-aminoacyl-tRNA-deacylase (DTD), which usually hydrolyzes d-aminoacyl-tRNAs, was inactive against d-Ala-tRNA^Ala^. To support the finding about DTD, computational docking and molecular dynamics simulations were run. Overall, our work illustrates the novel function of the AlaRS editing domain in stereospecificity control during translation together with *trans*-editing factor DTD. Thus, we propose different evolutionary strategies for the maintenance of chiral selectivity during translation.

## INTRODUCTION

The strict chiral prevalence in biological molecules is displayed in every single organism on Earth. In contrast to living species, organic materials in the universe are found in a racemic mixture (1,2). In this context, an organism-independent mixture of amino acids found in the Murchison meteorite demonstrated that alanine (Ala) and achiral glycine (Gly)—the simplest amino acids—had the highest abundance (3). Moreover, the d/l-Ala molar ratio was shown to be almost 50/50 (4). Homochirality of amino acids is essential for natural protein biosynthesis, and only l-enantiomers are present in proteins. Probably, the selectivity of l-amino acids was determined by the stereochemistry of RNA (5).

Nevertheless, the role of free d-amino acids in bacteria and in eukaryotes is diverse: from their involvement in signaling pathways (6–9) to building molecules of peptidoglycan in cell walls (10–12). Notably, the cellular concentration of l/d-stereoisomers varies from nano- to micromolar in eukaryotes (13) and can reach millimolar concentrations in some prokaryotes (14). Interestingly, the millimolar levels of d-Ala and d-Glu, the main components in peptidoglycans of bacterial cell walls, are higher than the levels of their l-isomers (10).

Recent data show that a prevalence of one amino acid enantiomer may lead to toxicity for a different one (15). Intensive studies of d-amino acids’ role discovered an effective enzymatic racemization mechanism in the archaeon, *Methanococcus maripaludis* (16) and in a yeast, *Schizosaccharomyces pombe* (17), leading to a conversion of d-alanine (d-Ala) to l-alanine (l-Ala), which is an unusual use of the d-enantiomer as a nitrogen source. Thus, the free d-amino acids in cell cytosol are potentially essential for both pro- and eukaryotic systems, despite the fact that only l-amino acids are used in protein biosynthesis.

Aminoacyl-tRNA-synthetases (aaRSs) activate amino acids and attach them to cognate tRNAs in a two-step reaction: (i) the formation of aaRS–aminoacyl–AMP complex by releasing the pyrophosphate; (ii) the binding of specific tRNA to aaRS-aminoacyl by AMP release and, finally, the creation of the covalent bond between cognate amino acid and tRNA, better known as ‘charged’ or aminoacyl-tRNA (18). These unique enzymes represent a large family with a diverse structural organization and have been divided into two classes (19–21). Alanyl-tRNA-synthetase (AlaRS), the class II member, is known to be one of the most stable members of this family (together with GlyRS, LeuRS, IleRS and ValRS) (22). Tyrosyl-tRNA-synthetase (TyrRS), belonging to class I, is a late member in the biosynthetic machinery (23). AlaRS can easily misactivate both the smaller amino acid Gly and the larger – l-serine (l-Ser) and hydrolyze them (24) by editing domain. The current state of the mechanism of chiral discrimination during the aminoacylation step is defined by the active site of aaRS (25). Lacking an editing domain, TyrRS effectively attaches both enantiomers of tyrosine (l-Tyr and d-Tyr) (26), but this problem is solved by d-aminoacyl-tRNA-deacylase (DTD), the *trans*-editing factor, which breaks the ester linkage between some d-amino acids and tRNA (26,27).

Here, we studied the stereospecificity of two different aaRSs with respect to amino acid in their evolutionary context: AlaRS (one of the earliest aaRSs) and TyrRS (one of the latest ones) from *Thermus thermophilus* (phylum *Deinococcus-Thermus*). These enzymes fundamentally differ in the level of structural-evolutionary honing of their active center according to the structure of a homologous amino acid (l-Tyr or l-Ala): from highly specific (in the case of TyrRS) (28) to relaxed mode (in the case of AlaRS) (29). In the current study, we compared the mechanisms used by *T. thermophilus* AlaRS and TyrRS to achieve full specificity as the distinct members of the aaRSs. We analyzed the catalytic velocities of cognate (l-Ala or l-Tyr) and non-cognate amino acid (d-Ala, Gly, l-Ser, d-Ser or d-Tyr) activation by AlaRS and TyrRS and checked the editing activity of DTD and AlaRS against noncognate misacylated substrates Gly/d-Ala-tRNA^Ala^. It turned out that DTD effectively hydrolyzes both d-Tyr-tRNA^Tyr^ and Gly-tRNA^Ala^, but it is not active against d-Ala-tRNA^Ala^. On the other hand, we discovered evidence of the misbinding of d-alanine, its attachment to cognate tRNA^Ala^ and hydrolysis by AlaRS, which was previously unknown. Together with molecular dynamics (MD) simulations and kinetics data, we propose an evolutionary strategy for the chiral selectivity of the translation apparatus.

## MATERIALS AND METHODS

### AlaRS and DTD expression and purification

*Thermus thermophilus* AlaRS (AlaRSTT) (NCBI accession number AAS81822.1) was expressed in BL21(DE3)Star *Escherichia coli*. A His-tagged protein construct was created and the gene was ligated into the pET28b vector. After induction with 1 mM isopropyl β-D-1-thiogalactopyranoside (IPTG), the culture was grown for 3 h at 37°C. The details of the purification procedure, including stepped affinity and size-exclusion chromatography, were described in our recent paper (30). All data about *T. thermophilus* DTD (DTDTT) expression and purification were delineated in this article (31), and a protocol for site-directed mutagenesis of the DTDTT gene was mentioned here (32). The plasmid, containing the gene of mutant *E. coli* C666A AlaRS, was kindly given to us by Prof. P. Schimmel (Scripps Research Institute, La Jolla, CA). This protein was expressed and purified according to the previously developed procedures (24).

### tRNA^Ala^ cloning, synthesis and labeling

tRNA^Ala^ was purified *in vivo* from BL21(DE3)Gold *E. coli* cells, harboring a pET9a-tRNA^Ala^ expression plasmid. The gene encoding *T. thermophilus* tRNA^Ala^ (NC_006461.1) was cloned into a pJET 1.2 vector, followed by transformation of *E. coli* Top10, restriction and sequencing. Two primers were designed (5′-**GTCGAC**TAATACGACTCACTATAGG-3′ and 5′-**GGATCC**TGGTGGAGCCGAGGGGATTCG-3′), containing the restriction sites for SalI and BamHI, respectively. After successful confirmation of cloning by sequencing, the tRNA^Ala^ construct was ligated with the pET9a expression vector. *E. coli* BL21(DE3)Gold cells were transformed by electroporation (BioRad system) and further grown in 2xTY medium, supplemented with 50 μg/ml kanamycin and 10 μg/ml tetracycline. tRNA^Ala^ expression was induced by 0.5 mM IPTG and proceeded for 14 h at 37°C. The total tRNA extract was obtained. tRNA^Ala^ was purified to homogeneity on an ion-exchange column with Q-Sepharose (Bio-Scale™ Mini UNOSphere Q, GE Healthcare), then on a DEAE 5PW column, followed by ion chromatography on C3 (Beckman). Purity was verified using 8% urea-containing polyacrylamide gel electrophoresis; activity was tested in aminoacylation by AlaRSTT with 20 μM radiolabeled [^14^C]-Ala (158 mCi/mmol, Amersham).

CCA-cutting was followed by 0.005 U/ml phosphodiesterase I from *Crotalus adamanteus* venom (Sigma) for 45 min at room temperature in 50 mM Tris–HCl (pH 8.0), 10 mM MgCl_2_ and 20 μM tRNA^Ala^*in vivo* construct. Phenol–chloroform extracted and ethanol precipitated tRNA^Ala^ was purified using high-pressure liquid chromatography (HPLC) on ProSwift™ Wax–1S column (DIONEX). The column was pre-equilibrated with 200 mM NaCl in buffer A (20 mM Tris–HCl (pH 7.5), 8 mM MgCl_2_, 10% v/v 2-propanol). tRNA was eluted with the programmed linear gradients of buffer B (1 M NaCl in buffer A) and ethanol precipitated.

Synthesis of [^32^P]-A76 tRNA^Ala^ was performed with 150 NTase, 10 μM tRNA^Ala^ (lacking CCA-end) and 0.8 μM [^32^P]-ATP (3000 Ci/mmol; Perkin Elmer) for 5 min at 60°C according to the recommendations in (33). The sample was purified using 8% urea-containing denaturing polyacrylamide gel, ethanol precipitated and refolded. Oxidized tRNA^Ala^ or tRNA^Ala^_Ox_, (for controls in AMP accumulation tests), was produced by incubation of 3 μM tRNA^Ala^ in the dark in 100 mM NaOAc (pH 5.02), 3 mM NaIO_4_ (Sigma) at room temperature for 2 h. The incubation with ethylene glycol was performed to eliminate the excess NaIO_4_ as described in (34).

### AlaRS and TyrRS ATP-PPi exchange assays

ATP-PPi exchange by AlaRSTT was performed at 60°C in AlaRS reaction buffer consisting of 75 mM HEPES-NaOH (pH 7.5 at 25°C), 10 mM MgCl_2_, 20 mM KCl, 1 mM dithiothreitol (DTT), 4 mM adenosine triphosphate (ATP) and supplemented with 1 mM [^32^P]-PPi (5 μCi/ml; Perkin Elmer). The concentrations of l/d-alanine, glycine, l/d-serine (Sigma) varied over the range 0.07–14 *K*_m_ (l-Ala), 0.05–11 *K*_m_ (d-Ala), 0.23–11 *K*_m_ (Gly), 0.05–10 *K*_m_ (l-Ser) and 0.12–2.5 *K*_m_ (d-Ser). The highest concentration of d-Ser (2.5 *K*_m_, 1 M) was limited by its low solubility. Reactions were initiated by the enzyme with different concentrations for each substrate. Reaction aliquots of 1.5 μl were removed at time points, quenched with cold 400 mM NaOAc (pH 5.02), treated with S1-nuclease (ThermoScientific) and followed thin layer chromatography (TLC) analysis according to (35). At least three independent measurements were averaged to determine kinetic parameters (*K*_m_ and *k*_cat_ values) from a Michaelis–Menten plot using OriginPro 8.5. The TLC plates were quantified by phosphor-imaging using PharosFX™ Plus System and QuantyOne software (BioRad).

The TyrRS reaction buffer was the following: 25 mM HEPES-NaOH (pH 7.5), 10 mM MgCl_2_, 15 mM KCl, 5 mM DTT. Concentrations of ATP and [^32^P]-PPi were the same as mentioned above for AlaRS. l-Tyr (Pierce) and d-Tyr (Sigma) varied over the range 0.11–11 *K*_m_ and 0.04–18 *K*_m_, respectively.

### AlaRS aminoacylation, deacylation and overall editing assays

Aminoacylation assays with radiolabeled tRNA^Ala^ and cognate/noncognate amino acids for AlaRS were performed at 37°C in standard AlaRS buffer in the same conditions as those mentioned above for AMP accumulation reactions.

Preparative amounts of l-Ala-[^32^P]-tRNA^Ala^ were obtained during 15 min at 37 °C in the AlaRS standard buffer, supplemented with 1 mM l-Ala, 100 nM AlaRSTT, 10 μM tRNA^Ala^ and 4 mM ATP. Misaminoacylated d-Ala-tRNA^Ala^ and Gly-tRNA^Ala^ were produced using an editing-deficient *E. coli* C666A mutant form of AlaRS. d-Ala-[^32^P]-tRNA^Ala^ was generated for 25 min at 37 °C (500 mM d-Ala, 5 μM *E. coli* C666A AlaRS, 10 U/ml inorganic pyrophosphatase from baker's yeast—PPiase); Gly-[^32^P]-tRNA^Ala^—20 min at 37 °C (750 mM Gly, 500 nM *E. coli* C666A AlaRS, 2 mM ATP). The aminoacylation reaction was followed by acidic phenol extraction and ethanol precipitation of aminoacylated tRNA^Ala^. At last, the aminoacylated tRNA^Ala^ was resuspended in 20 mM NaOAc (pH 5.02) and kept at –20°C. The yields of l/d-Ala/Gly-tRNA^Ala^ were estimated using TLC analysis following the treatment of the tRNA sample with S1 nuclease. All aminoacylated tRNA^Ala^ resulted in ∼30% level of substrate formation.

The overall editing activity was determined by measuring [^32^P]-AMP formation at 60 °C for 30 min in a standard AlaRS reaction buffer supplemented with 1 mM ATP, 2 mM l-Ala or 50–500 mM d-Ala/Gly/l-Ser/d-Ser and traces of [^32^P]-ATP (3000 Ci/mmol) and 100 μg/ml bovine serum albumin (BSA) in the absence or in the presence of 10 μM tRNA^Ala^ or tRNA^Ala^_Ox_ and 1 μM AlaRSTT. Reactions were initiated by addition of 5 × enzyme, stopped by quenching a 1.5 μl aliquot in 3 μl of 400 mM NaOAc, followed by S1-nuclease digestion (1.5 μl of an aliquot in 3 μl of 4 U/μl solution of S1) and TLC analysis.

Deacylation assays were performed with 0.25 μM l-Ala/d-Ala/Gly-[^32^P]-tRNA^Ala^ at 37 °C in standard AlaRS reaction buffer or standard DTDTT buffer (31) supplemented with 0.1 mg/ml BSA. Enzyme concentrations were varied. Reaction mix at various time points was subjected to S1-nuclease treatment and analyzed as described above.

### Computer modeling of AlaRSTT in complex with l-Ala-/d-Ala-/l-Ser-/d-Ser-AMP and DTDTT in complex with l-Ala/d-Ala-tRNA^Ala^

A homology model of AlaRSTT was generated using Swiss-Model Workspace (36) based on the BLAST (37) sequence alignments and known X-ray structures from the RCSB protein databank (https://www.rcsb.org/) (38,39). A homodimer structure was rebuilt in PyMol (40) (The PyMOL Molecular Graphics System, Version 1.5.0.4 Schrödinger, LLC), which was also the main tool for graphical representation.

The aminoacylation site was identified from the spatial alignment of the crystal and homology proteins. A relaxation period of 50 ns was set to reach satisfactory root-mean-square deviations (RMSD) fluctuations. MD simulations of the ligand-free protein structure were executed and analyzed using GROMACS 4.6.3 software (41). Each of the ligand topologies was generated using the Antechamber program (42). A ligand-binding site of the protein with each compound was obtained from the clustering procedure and selection of the centroid conformation via in-built GROMACS tools. Docking of aminoacyl-adenylates and aminoacyl-tRNA fragments was performed using GOLD CCDC (43) applying all standard settings by default; the rearrangement of ‘flipping mode’ for the carboxylic group on ‘rotation mode’ and the introduction of the ‘ASP’ function of a rescoring protocol was changed. Finally, the docking was carried out with the same input parameters used in our previous study (32). The settings for MD simulation of l- and d-stereoisomers, which should be comparable with those conditions for DTD were also the same.

### MD calculations of l-Ala- and d-Ala-tRNA^Ala^ editing substrates in DTDTT active site

To estimate the probability of the water attack on the l- and d-alanyl-tRNA^Ala^ fragments, the MD trajectories were analyzed so as to avoid a human factor. For this reason, all the calculations on the identification of the potential prereaction states in the deacylase complexes were performed with a self-developed Python script (Supplementary additional data S1). The idea for this script was inspired by known mechanisms of nucleophilic attack and required conditions for its occurrence. First of all, the trajectory of the MD was processed with in-built GROMACS tools to derive the ligand, all surrounding amino acid residues and those water molecules that were found at 3.5 Å at least once during the MD simulation. Then, an output file that contained 5000 frames was read line by line with the extraction of each frame and identification of those water molecules that met the distance (<3.5 Å) and Burgi–Dunitz angle (105–107°) conditions. The water molecule orientation plays an important role. The orientation was determined by comparison of three distances between the carbonyl carbon of the ligand and each atom of the water molecule to ensure that the oxygen of the water is closer than other atoms.

In the case of a success, an assistant molecule search was executed by estimation of the distances and orientation between other water molecules and the attacking one. If any water molecule was located within 3.5 Å of the attacking water molecule, the space around was examined to detect amino acid atoms, which could activate or, at least, fix the assistant water molecule. All step-by-step derived results were written into the data list.

## RESULTS

### *T. thermophilus* AlaRS misactivates d-Ala and d-Ser

Being distinct members of the aaRSs family, TyrRS and AlaRS may have different specificity to d-amino acids. TyrRS has the weakest stereospecificity among all aaRSs (26,44), thus, it attaches both enantiomers of Tyr with similar catalytic activity. Based on pre-steady state kinetics data, Sheoran *et al.* observed that side chains of l-Tyr and d-Tyr bind to *Bacillus stearothermophilus* TyrRS in almost identical way that is essential for catalytic attack (45). Recently, we showed that the 2′-OH group of terminal adenosine (A76) of tRNA^Tyr^ is essential for the control of d-Tyr mistranslation (31). Here, we measured the level of substrate specificity in ATP-PPi exchange assay by *T. thermophilus* TyrRS (TyrRSTT). In comparison with kinetic parameters for the aminoacylation reaction (31), *K*_m_ for l-Tyr and d-Tyr in the amino acid activation step had only a 2.4-fold increase (9 ± 2 μM versus 3.7 ± 0.35 μM) for l-Tyr and 5.2-fold increase (27.6 ± 7.5 μM versus 5.3 ± 0.5 μM) for D-Tyr. The discrimination factor, representing the substrate specificity of enzyme, did not differ significantly during the aminoacylation reaction: 24 at the first step, the aminoacyl-adenylate formation and 19 at the second step, the aminoacyl-tRNA formation (Table 1). Thus, TyrRS showed very low stereoselectivity in recognition of Tyr enantiomers. Lacking an editing domain, it cannot correct these mistakes itself; thus DTD, *trans*-editing factor, exists to overcome this problem and hydrolyze misacylated d-Tyr-tRNA^Tyr^ complexes (31,32).

**Table 1.** Steady-state kinetics for aminoacylation by TyrRSTT

Tyr	*k*_cat_ (s ^–1^)	*K*_m_ (μM)	*k*_cat_/*K*_m_ (μM ^–1^ s ^–1^)	Discrimination factor
ATP-PPi exchange assay				
l-Tyr^a^	17.36 ± 3.9	9 ± 2	1.93	1
d-Tyr^b^	2.2 ± 0.65	27.6 ± 7.5	0.08	24
Aminoacylation assay				
l-Tyr^c^	46.7 ± 6.7	3.7 ± 0.35	12.76	1
d-Tyr^c^	3.5 ± 0.2	5.3 ± 0.5	0.66	19

The data shown represent mean values ± SEM (*n* = 3), measured by ATP-PPi exchange assay at 60 °C, pH 7.5.

^a^TyrRSTT was assayed at 5 nM concentration.

^b^TyrRSTT was assayed at 50 nM concentration.

^c^Data from (31).

Discrimination factor = (*k*_cat_/*K*_m_)_l-Tyr (cognate amino acid)_/(*k*_cat_/*K*_m_)_d-Tyr (noncognate)_.

In contrast to TyrRS, AlaRS has its own apparatus for fidelity control: (i) a central editing domain (24), homologous to the editing domain of the class II ThrRS (46); (ii) free-standing AlaX proteins (AlaXps) (types Ia, Ib, II or AlaX-S, AlaX-M and AlaX-L, standing for small, medium and large, respectively) (47,48), acting on tRNAs (tRNA^Ala^ or tRNA^Thr^ (47)), mischarged by Ser or Gly. The necessity of the wide range of *trans*- and *cis*-editing modules in the AlaRS system is caused by the high frequency in misactivation of noncognate amino acids (Gly, Ser).

Here, we checked the possibility of AlaRSTT to bind d-Ala and d-Ser and compared it with activation rates for other amino acids—l-Ala, l-Ser and Gly (Table 2). In addition, we tested our hypothesis on the well-studied *E. coli* C666A AlaRS editing-deficient mutant enzyme (Supplementary Table S1). Compared with l-Ala, the value of *k*_cat_ decreased by 38-fold and *K*_m_ increased 16-fold for d-Ala, which indicated that the enzyme required more substrate to reach half-saturation. In contrast, *k*_cat_ for Gly (6.5 ± 1.6 s^–1^) is similar to l-Ala (7.88 ± 2.6 s^–1^). The value for l-Ser had a 3-fold decrease (2.3 ± 0.8 s^–1^), compared with l-Ala. d-Ser showed the lowest catalytic efficiency: *k*_cat_ decreased 62-fold, but *K*_m_ demonstrated a 2930-fold increase, illustrating an extremely low affinity of the substrate.

**Table 2.** Kinetic constants of AlaRSTT in amino acid activation reaction

Amino acid	*k*_cat_ (s^–1^)	*K*_m_ (mM)	*k*_cat_ /*K*_m_ (mM^–1^ s^–1^)	Discrimination factor
l-Ala^a^	7.88 ± 2.6	0.139 ± 0.012	56	1
d-Ala^b^	0.21 ± 0.03	2.19 ± 0.50	0.12	467
Gly^c^	6.5 ± 1.6	22 ± 8	0.29	193
l-Ser^d^	2.3 ± 0.8	10 ± 2	0.236	237
d-Ser^e^	0.127 ± 0,028	407 ± 59	0.00031	180 645

The data shown represent mean values ± SEM (*n* = 3), measured by ATP-PPi exchange assay at 60 °C (pH 7.5).

^a/c^AlaRSTT was assayed at 15 nM concentration.

^b^AlaRSTT was assayed at 1 μM concentration.

^d^AlaRSTT was assayed at 100 nM concentration.

^e^AlaRSTT was assayed at 2.5 μM concentration.

**Figure 1.**
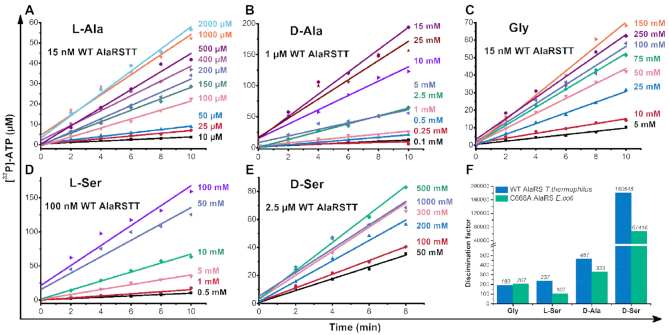
Kinetics of amino acids activation in ATP-PPi exchange assay. Activation of l-Ala (**A**), d-Ala (**B**), Gly (**C**), l-Ser (**D**), d-Ser (**E**); rates of substrate specificity (discrimination factor), measured by AlaRSTT (blue bars) and *Escherichia coli* C666A AlaRS (green bars) (**F**). On the right of the graphs A–E, the appropriate concentrations of amino acids are indicated.

The global effect corresponded to a 193-fold loss in catalytic efficiency for Gly, 237-fold—for l-Ser, 467—for d-Ala and ∼180 650-fold for d-Ser (Table 2). Similar rates were obtained for *E. coli* C666A AlaRS (Supplementary Table S1): 107-fold loss in efficacy for l-Ser, 207 for Gly, 333 for d-Ala and 67 400 for d-Ser. Kinetics rates, measured in ATP-PPi exchange over time, shown in Figure 1. Original TLC plates, displaying time-course activation of d-Ala and d-Ser by AlaRSTT, are shown in Supplementary Figure S1. The comparison of our data with literature is shown in Supplementary Figure S2, representing a general trend for three systems.

To investigate the kinetics data further, we performed an MD simulation of l-Ala/d-Ala-AMP and l-Ser/d-Ser-AMP, bound to AlaRSTT. A homology model was built using a pair of template structures (PDB ID: 3HTZ, 3WQZ) with a different degree of similarity (32–58%) to the target protein (UniProt: P61707). Next, the two derived models were combined after a structural alignment and mutual replacements of the low-quality fragments (missed or disordered regions, generated due to the low-level similarity threshold). This homodimer structure was relaxed during 25 ns of a free MD simulation.

In general, the analysis of the trajectory showed the entire stability of the structure. However, one loop (NYWPGGAITHGPNGPSG) near the amino acid binding site could be important for the binding step. To understand how the selectivity is provided, a series of MD simulations with l- and d-stereoisomers of alanyl- (Ala-AMP) and seryl-adenylates (Ser-AMP) was conducted. To generate a reliable complex of AlaRSTT with adenylates, the most stable and voluminous shape of the binding site was found. The clustering analysis of the MD trajectory determined five of the most frequent conformations, but only two of them possessed opened binding site geometry and were suitable for molecular docking.

Thus, the initial protein structure geometry was common for all four complexes; the orientation of adenine moiety and the interaction map of all ligands were similar to those from the source structure (PDB ID: 3HXY). The protein component was represented with a truncated monomer, lacking the last 160 residues. The result of the MD simulation revealed a notable correlation between *in vitro* and *in vivo* trends. For example, the prevalence of l-isomers over d-forms is evident from the inspection of interaction energy values (Supplementary Figure S4F) and H-bond number plots, especially for l-alanyl-adenylates (Supplementary Figure S4F). The RMSD graph comparison showed the significant stability of l-alanyl-adenylates and a decrease of these indices for other ligands (Supplementary Figure S4A–C).

**Figure 2.**
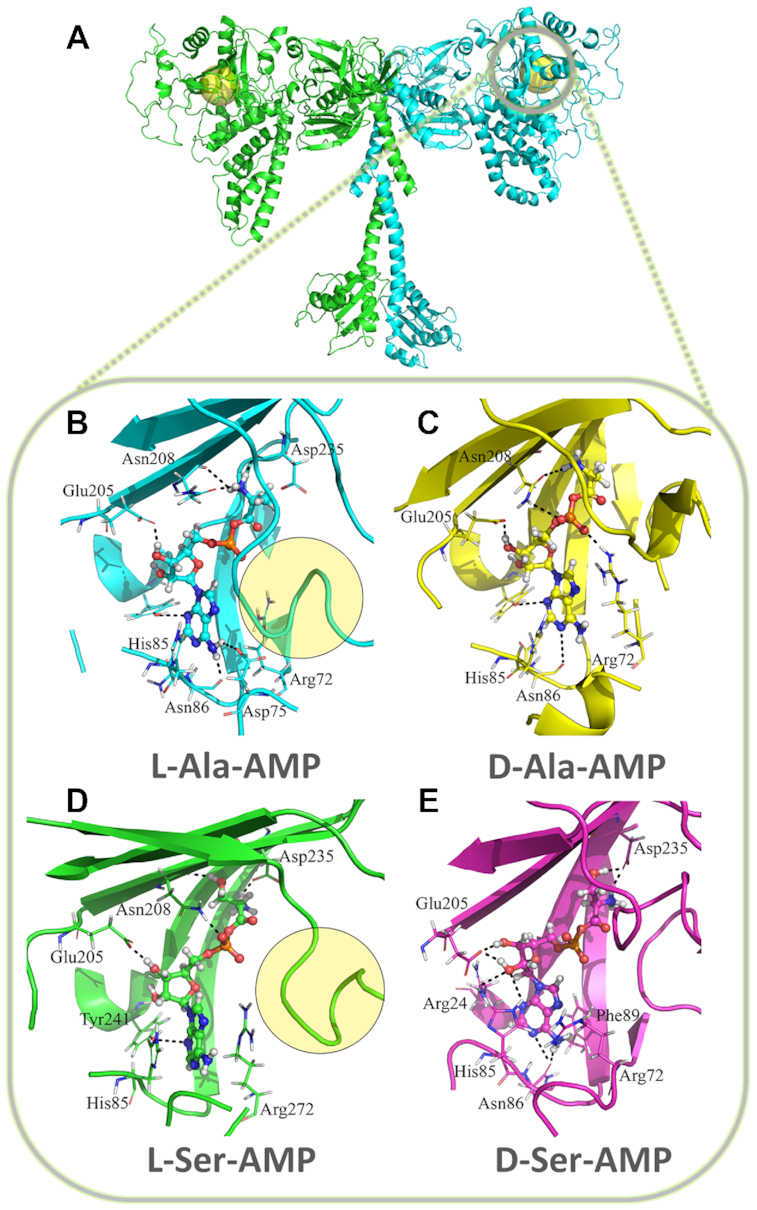
Details of AlaRSTT active site with cognate and non-cognate substrates. (**A**) A generalized state of all studied aminoacyl-adenylates, corresponding to the final frame of MD simulations of AlaRS. It demonstrates some similarity in the position of the substrate for l-Ala-AMP (**B**) and l-Ser-AMP (**D**) and the decrease in a donor–acceptor pair number and disappearance of a favorable mutual orientation, contributing to the stacking between purine and Arg72, His85 and Asn86 in d-Ala-AMP (**C**) and d-Ser-AMP (**E**). This trend is more pronounced in a set of graphs with interaction energy and H-bond number comparison (Supplementary Figures S3–4).

The general scheme of final MD simulations of AlaRSTT is shown in Figure 2A. Another interesting observation, which can be considered to be specific for the AlaRS, is the deformation of the loop, mentioned above, in the entrance of the binding site. Interestingly, that loop is much longer in AlaRSTT than in other homologues, and its conformation is distinct for l- and d-isomers (the position of the loop is indicated by the yellow circle on Figure 2B and D). Visual inspection of the final frames showed the extended H-bond network and a more compact, shield-like conformation of the loop for l-forms. We suggest that the motion of the loop is not a coincidence and it can either increase the ligand flexibility (d-isomers) or restrict the ligand stability (l-isomers). The presence of a stacking interaction between Arg72, His85 and Asn86 stabilizes the nucleotide moiety of aminoacyl-adenylates (Figure 2B–D), while Asp235 and Asn208 define the enhanced binding of the l-forms (Figure 2B and D).

### AlaRSTT mischarges tRNA^Ala^ with d-Ala and d-Ser

**Figure 3.**
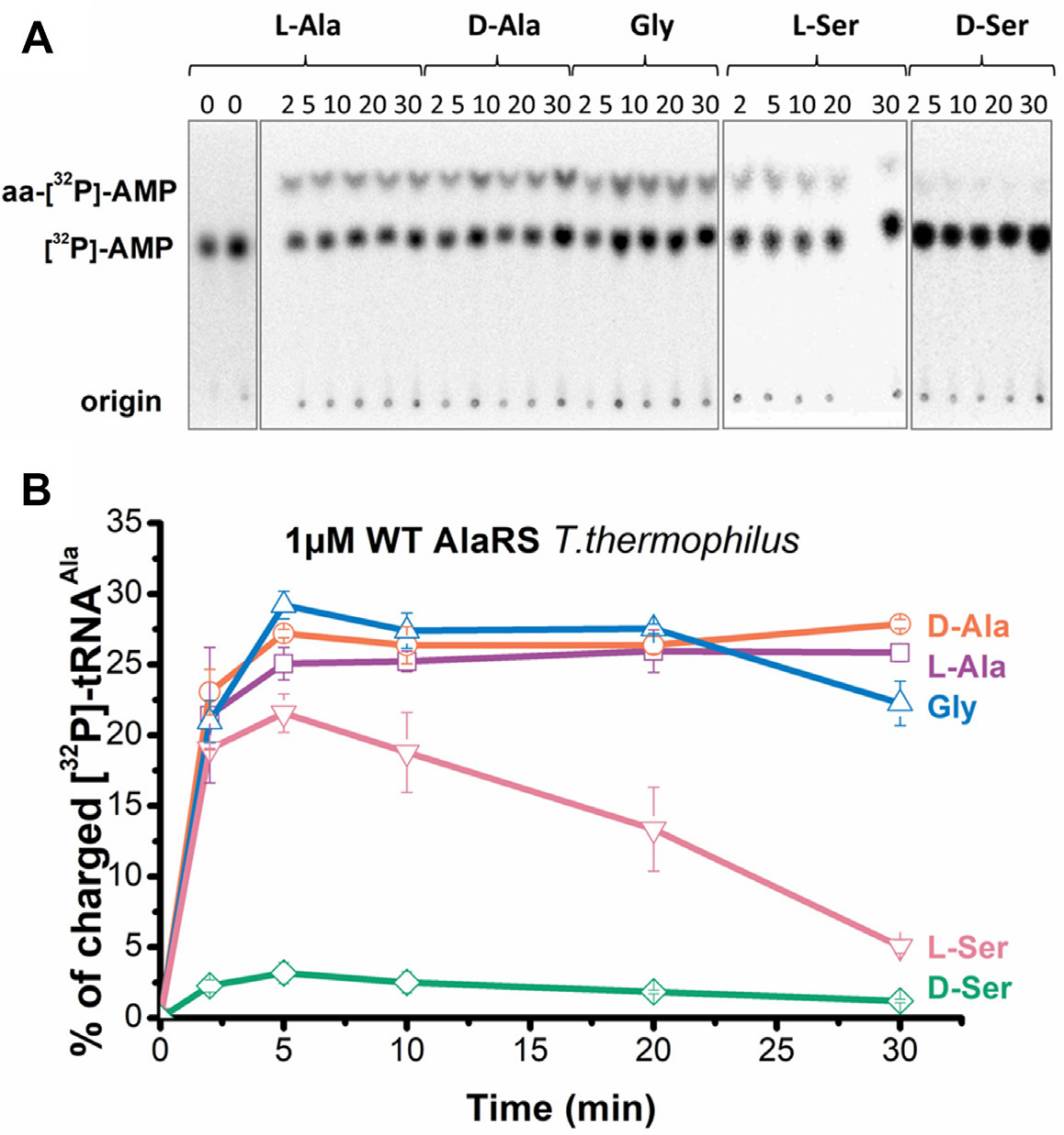
Charging of tRNA^Ala^ by WT AlaRSTT with l-alanine, d-alanine, glycine, l-serine and d-serine (+37 °C, pH 7.5). (**A**) TLC-based time-course aminoacylation assay showing chromatographic separation of aminoacyl-[^32^P]-AMP and [^32^P]-AMP. (**B**) Graphical representation of TLC data shown in panel A.

Taken together with the literature data from previous studies on AlaRS, we did not find enough evidence about actual rates of aminoacylation with all noncognate substrates. We performed aminoacylation assays with l-Ala, Gly, l-Ser, d-Ala and d-Ser and tested the potential charging of [^32^P]-tRNA^Ala^ by d-Ala and d-Ser. Interestingly, with purified *in vivo* tRNA^Ala^, we observed similar rates (∼30% of aminoacyl-tRNAs) for three amino acids: cognate l-Ala and noncognate Gly and d-Ala (Figure 3B). Figure 3A shows a TLC plate analyzing the time course of aminoacylation of [^32^P]-tRNA^Ala^ by 1 μM AlaRSTT using 2 mM nonradioactive l-Ala, 500 mM Gly/d-Ala/l-Ser/d-Ser. Saturated concentrations of both substrates were chosen (10 μM tRNA^Ala^). The aminoacylation rate for l-Ser decreased over time from 20 to 5% during the 30 min reaction. These data can confirm the high level of deacylation by AlaRSTT against Ser-tRNA^Ala^, which is also significant in *E. coli* (24,49). The analogous steady-state level of Ala-tRNA was observed during the aminoacylation of double-specific Phe/Ala-tRNA (YFA2) by *E. coli* AlaRS (50). In contrast to observations on *E. coli* AlaRS (50), the addition of inorganic PPiase did not influence the level of aminoacylated *T. thermophilus* tRNA^Ala^ (data not shown). Thus, our findings reflect the equilibrium state between aminoacylation and deacylation rates, catalyzed by AlaRSTT.

The kinetic parameters for the aminoacylation step were determined for l-Ala, d-Ala and Gly. In contrast to amino acid activation reaction, the value of *k*_cat_ decreased by 9-fold for l-Ala, 70-fold for d-Ala and 140-fold for Gly (Table 3). The *K*_m_ for d-Ala showed a 12-fold increase while for l-Ala and Gly this parameter did not significantly change. These results indicate the low affinity of noncognate amino acids in aminoacylation reaction with tRNA^Ala^ and the high requirement for hydrolysis of misacylated-tRNAs.

**Table 3.** Steady-state aminoacylation by AlaRSTT

Amino acid	*k*_cat_ (s^–1^)	*K*_m_ (mM)	*k*_cat_ /*K*_m_ (mM^–1^ s^–1^)
l-Ala^a^	0.86 ± 0.22	0.086 ± 0.020	10
d-Ala^b^	0.003 ± 0.0002	27.35 ± 8.01	0.00011
Gly^c^	0.045 ± 0.018	26.35 ± 7.99	0.00170

The data shown represent mean values ± SEM (*n* = 3), measured in aminoacylation with [^32^P]-tRNA^Ala^ at 60 °C (pH 7.5).

^a^AlaRSTT was assayed at 15 nM concentration.

^b^AlaRSTT was assayed at 1 μM concentration.

^c^AlaRSTT was assayed at 100 nM concentration.

### Misacylated d-Ala-tRNA^Ala^ was hydrolyzed by AlaRSTT, but not by DTDTT

To check the ability of AlaRS to hydrolyze the misactivated tRNA^Ala^, deacylation assays with [^32^P]-Gly/d-Ala-tRNA^Ala^, comparing with l-Ala-tRNA^Ala^, were performed with 500 nM AlaRSTT. In parallel, the activity of wild-type (WT) DTDTT and its Y125F mutant form was tested against mischarged tRNA^Ala^ with d-Ala. Compared with WT DTDTT, Y125F was found in our previous study with a 270-fold increase in catalytic velocity of deacylation d-Tyr-tRNA^Tyr^ (32). As a result, it was important to check its activity on d-Ala and l-Ala-tRNA^Ala^.

**Figure 4.**
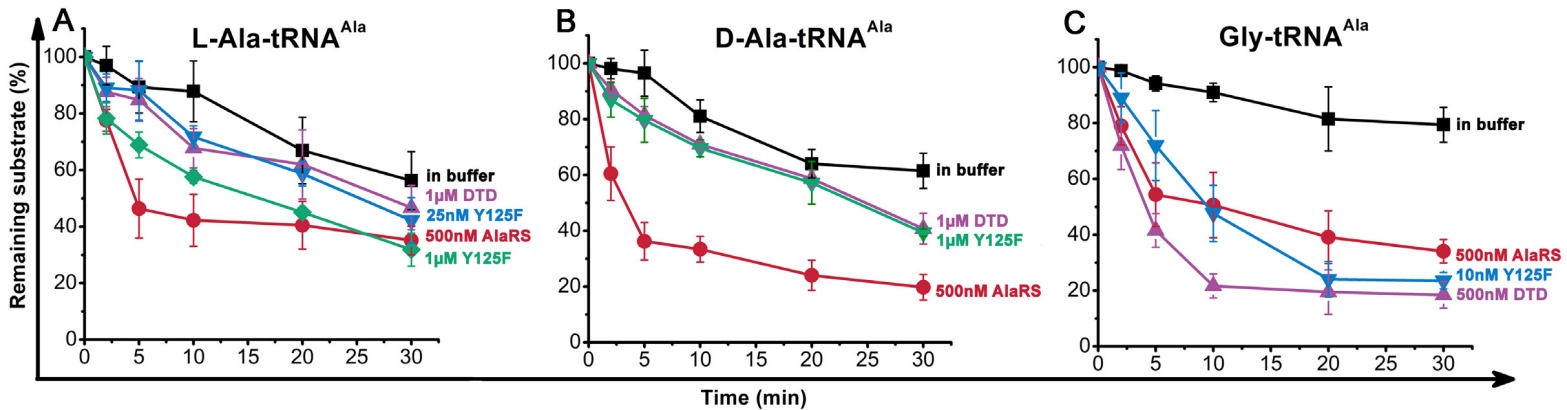
Deacylation rates of l-Ala-tRNA^Ala^ (**A**), d-Ala-tRNA^Ala^ (**B**), Gly-tRNA^Ala^ (**C**) by WT AlaRSTT and WT DTDTT and its Y125F mutant at 37 °C (pH 7.5). The concentration of all substrates was 0.25 μM. The data correspond to the mean values with standard error of measurement (SEM) from three independent experiments.

Figure 4 represents the time course of deacylation of all substrates. Surprisingly, AlaRSTT showed the highest rate of hydrolysis of d-Ala-tRNA^Ala^ (80% of substrate was hydrolyzed after 30 min) in contrast to the slightly lower rate for l-Ala/Gly-tRNA^Ala^ (60% of substrate was hydrolyzed after 30 min). We hypothesized that the editing domain of AlaRSTT is also responsible for the control of stereospecificity in the translation apparatus. In contrast to AlaRSTT, DTDTT (both the WT and its more active mutant form Y125F) did not show any hydrolytic activity against misacylated tRNA^Ala^ with d-Ala. However, the enzyme demonstrated prominent hydrolysis rates for Gly-tRNA^Ala^. This fact was first described for *Plasmodium falciparum* and *E. coli* DTDs (51,52). According to the deacylation of l-Ala-tRNA^Ala^, WT *T. thermophilus* DTD revealed a low level of hydrolysis even with 1 μM enzyme (∼10%); its 1 μM Y125F mutant displayed a 2-fold higher level. Depending on the structure of enantioselectivity motif (Gly-Pro), two different classes of DTD exist, Gly-*cis*-Pro (DTD) and Gly-*trans-*Pro (ATD, Animalia-specific tRNA-deacylase) (53). It was shown that deacylation of l-Ala-tRNA^Ala^ occurred only for the ATD class (mammalian, human, chicken, *Danio rerio* DTDs) (53) in contrast to the DTD class (*E. coli* enzyme) (52). Thus, our results are in line with the data in the literature.

**Figure 5.**
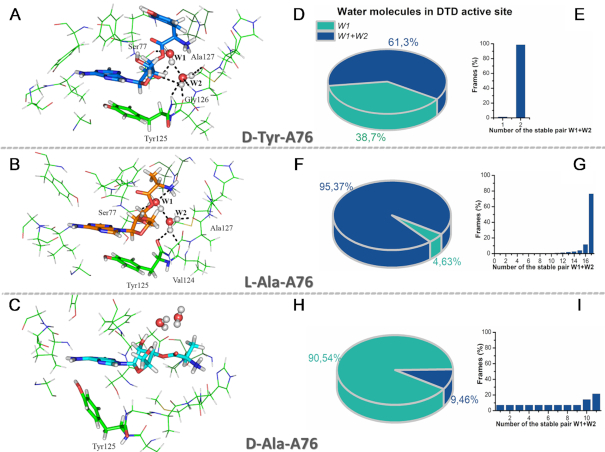
Spatial preconditions for the nucleophilic attack on different substrates in the DTD binding site. All complexes are represented with the same slice of MD (1370 ps), as was described for d-Tyr-A76 (**A**) in the paper (32). This frame reflects the overall trend for each simulation, namely for d-Tyr-A76 and l-Ala-A76 (**B**). In addition to the evident difference in the direction of attack, d-Ala-A76 (**C**) loses its stacking contacts with Tyr125 and forms a poor/weak H-bond network. (**D, F** and**H**) Representation of attacking (W1) and assisting (W2) water molecules in the active site of DTDTT during 5 ns MD simulations. (**E, G**and**I**) Number and rates of stable pair W1 with W2 over the time-course frames for d-Tyr-A76 (**E**), l-Ala-A76 (**G**), d-Ala-A76 (**I**).

To obtain more insights into deacylation of mischarged tRNA^Ala^ with l-Ala and d-Ala, we performed MD simulations of these substrates. The behavior of the substrates and the environment were analyzed with a self-developed script (described in the ‘Materials and Methods’ section) that identified all possible attacking (W1) and assisting (W2) water molecules in the active site of DTDTT during 5 ns MD simulations. In general, the substrate-assisted catalytic mechanism for the hydrolysis of l-Ala-tRNA^Ala^, provided by two water molecules, has much in common with those proposed for the hydrolysis of d-Tyr-tRNA^Tyr^ (32). Figure 5 shows preconditions for the nucleophilic attack of d-Tyr-A76 (A), l-Ala-A76 (B) and d-Ala-A76 (C). Water molecules that met the distance (<3.5 Å) and Burgi–Dunitz angle (105–107°) conditions were identified as W1. If an algorithm found an assistant water molecule within the appropriate distance (<3.5 Å) and potential amino acid residues that could activate it, the water molecule was labeled as W2 in the data list. In total, 124 frames were found to represent W1 in Burgi–Dunitz angle conditions for the hydrolysis of this substrate, and 76 of them (61.3%) also showed assisting molecules W2 (Figure 5D). In the time course of the MD simulation, only two stable pairs (W1+W2) were observed. One of this pair was anchored effectively and appeared in 98.7% of frames (Figure 5E). In contrast, for d-Ala-tRNA^Ala^, we obtained 148 frames in total, 14 of which also displayed W2 (9.5% of frames) (Figure 5H). Unfortunately, among 14 pairs of W1+W2, only one was presented on 21% of frames in the MD (Figure 5I). We hypothesize that the pure water environment disabled successful nucleophilic attack of the d-Ala-A76 substrate and resulted in no hydrolysis of DTDTT (Figure 4B). However, 782 frames of 820 in total (95.37%) represented the second water (W2) for l-Ala-tRNA^Ala^ hydrolysis by DTDTT (Figure 5F). Interestingly, among 17 stable water pairs, there was one that was present in 76% of frames (Figure 5G). This could explain the low hydrolysis rate of charged tRNA^Ala^ with l-Ala (Figure 4A).

Our findings demonstrated the high efficiency of AlaRSTT in the hydrolysis of misactivated tRNA^Ala^ with d-Ala in contrast to l-Ala. Compared with AlaRS, DTDTT exhibited more specificity to Gly-tRNA^Ala^ and did not hydrolyze d-Ala-tRNA^Ala^. It is noteworthy that the fastest nonfermentative hydrolysis of substrates is observed for l-Ala-tRNA^Ala^. Therefore, it appears that the AlaRS editing domain is also responsible for controlling the stereospecificity in protein biosynthesis machinery.

### Overall editing rates by AlaRSTT

**Figure 6.**
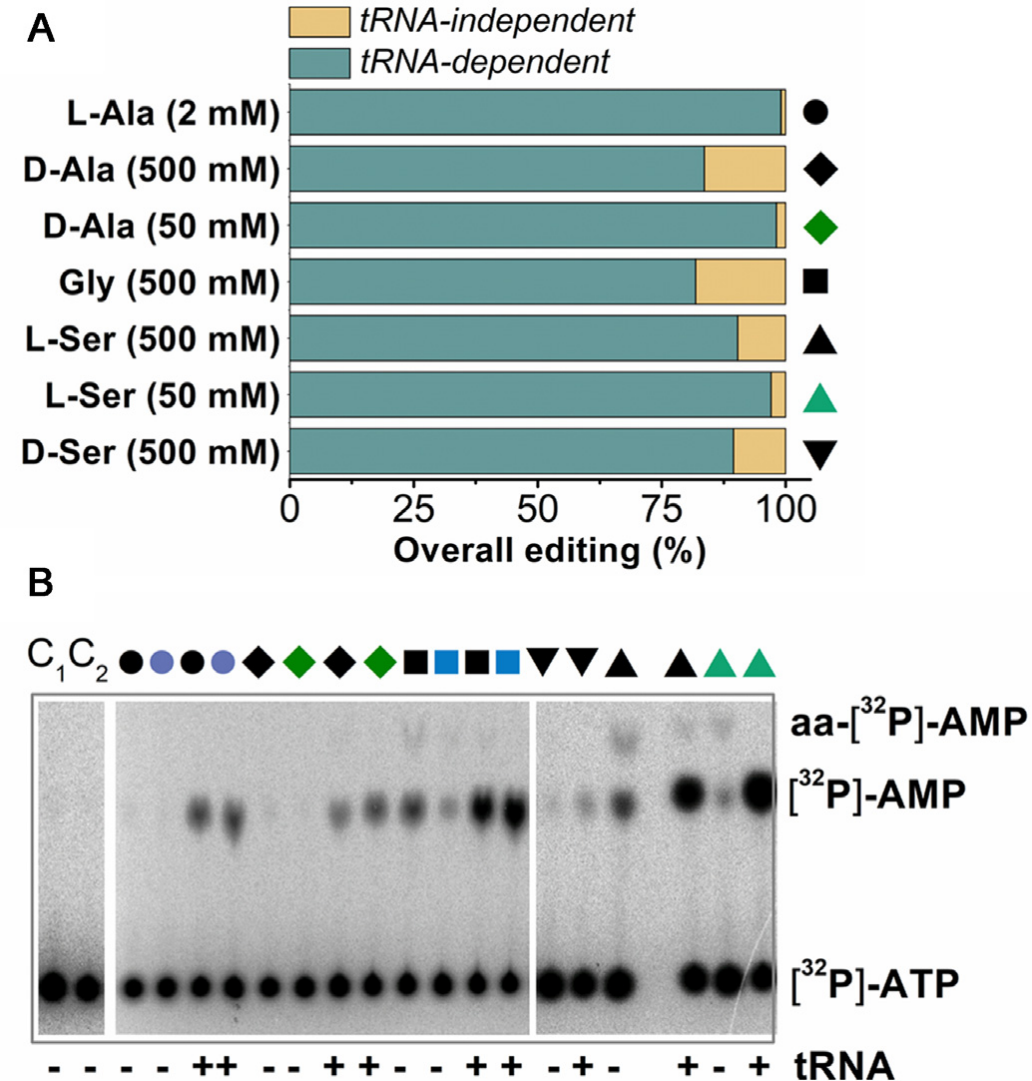
Formation of AMP by WT AlaRSTT (1 μM) at 37 °C (pH 7.5). (**A**) total level of AMP, accumulated after 30 min; (**B**) TLC of product formation, which was quantified in (A). Shapes in panels A and B mean the following: black circles–2 mM l-Ala, violet circles–0.2 mM l-Ala, black rhombs–500 mM d-Ala, green rhombs–50 mM d-Ala, black squares–500 mM Gly, blue squares–50 mM Gly, inverted black triangles–500 mM d-Ser, black triangles–500 mM l-Ser, green triangles–50 mM l-Ser. The final results represent the mean value of triplicate measurements.

Along with post-transfer editing, which occurs in a special editing domain of aaRS, the enzyme may directly hydrolyze the noncognate aminoacyl-adenylate via an inherent posttransfer editing mechanism for a catalytic site. To study the contribution of pre- and posttransfer editing pathways, we tested AlaRSTT in [^32^P]-AMP accumulation assays with l-Ala, d-Ala, Gly, l-Ser and d-Ser. Predictably, for all substrates, tRNA-dependent editing pathways predominated (Figure 6). tRNA-independent editing for l-Ala corresponded to approximately 1%, for d-Ala (50 and 500 mM, respectively) from 2 to 16 ± 6%. Similar rates of about 18 ± 4% were observed for Gly and 3 ± 0.7% for 50 mM l-Ser.10 ± 2% of tRNA-independent editing was demonstrated for both l-Ser and d-Ser (500 mM). To check the exact effect of tRNA in the overall editing assay, we performed a time-course AMP accumulation with oxidized tRNA^Ala^ (tRNA^Ala^_Ox_) by NaIO_4_ (Supplementary Figure S5). We conclude that tRNA is essential for the correct editing of misbound noncognate amino acids, which implies the possibility of a preferential posttransfer editing pathway of d-alanine by AlaRS. On the other hand, the weak tRNA-independent activity of AlaRS (probably, pretransfer editing) can be explained by the fact that the absolutely conservative W160, acting as a shield, protects the carboxyl of aminoacyl-adenylates from the nucleophilic attack and hydrolysis by water molecules. However, more studies are needed to determine the proper rates of tRNA-dependent/independent editing pathways.

## DISCUSSION

Here, we compared the potency of stereospecificity control in protein biosynthesis by evolutionarily early (AlaRS) and late (TyrRS) representatives of the aaRSs family (Tables 1 and 2; Figure 1). Both theoretical and experimental studies on aaRSs confirm the hypothesis of coevolution of the genetic code. Accordingly, the earliest (or starting) point was the code for Ala with the further addition of Gly and other amino acids; Tyr appeared among the group of amino acids that were added to the genetic code more recently (54). The earliest eight amino acids (alanine, aspartate, glutamate, glycine, isoleucine, leucine, proline and valine) (55), synthesized by Miller's electric discharge synthesis (56,57), were also found in the Murchison meteorite (58). Both l- and d-enantiomers of those eight amino acids were credibly involved in the earliest synthesis of primitive proteins built from amino acids in the environment before the evolution of biosynthetic pathways. The ancient emergence of Ala stimulates the translation apparatus to solve the paradox of potential misactivation of d-enantiomers (59). The convergence of early and late amino acids during evolution resulted in the different strategies of quality control during translation. For example, AlaRS possesses an editing domain, and TyrRS lacks one through evolutionary progress. It is probable that the emergence of DTD enzymes was promoted by a deficiency of TyrRS stereospecificity.

Notably, AlaRS shares difficulties in distinguishability between the smallest amino acids—glycine, alanine and serine. So, AlaRS can effectively charge tRNA^Ala^ with these small amino acids (60) and edit these misacylated substrates (24). However, pneumococcal AlaRS was also reported to misactivate tRNA^Phe^ (61). Our data revealed the new phenomena of d-amino acids activation and editing. *Thermus thermophilus* AlaRS was found to mischarge tRNA^Ala^ with noncognate d-Ala and d-Ser (Figure 3 and Table 3). Previously, very low aminoacylation rate only for d-Ala-minihelix (Supplementary Figure S2 in (5)) was shown. Authors assumed that homochirality in proteins was determined in the aminoacylation step and that the homochirality of RNA determined the selectivity of enantiomers. We hypothesize that different scenarios in the translation apparatus may exist to regulate the stereospecific control of amino acids depending on the propensity to lose the editing function of aaRS.

To correct the mistakes of non-cognate amino acid incorporation, two distinct groups of editing factors appeared in the process of evolution. The *cis-*editing function is maintained by the AlaRS editing domain, which is mainly responsible for both Gly and Ser-tRNA^Ala^ deacylation (24). AlaX-S is a *trans*-editing factor, which was found to have a lack of RNA specificity and to hydrolyze Ser-tRNA^Ala^/tRNA^Thr^/tRNA^Pro^ (47). ATD was reported to display relaxation of substrate specificity, deacylating l-Ala-tRNA^Ala^/tRNA^Thr^ (53) and DTD itself, demonstrating a positive selection of universal invariant tRNA^Ala^-specific G3:U70. ATD is a cellular glycine deacylator, hydrolyzing misactivated Gly-tRNA^Ala^ across all pro- and eukaryotes (51). In addition, two families of tRNA-dependent transferases, MurMN and FemABX exist to use l-Ala-tRNA^Ala^ as substrates for l-Ala transfer to a peptidoglycan cross-bridge. MurM is involved in the addition of the first amino acids (Ala or Ser) to the cross-bridge, MurN—of the second amino acid (Ala) (62) to lipid intermediate II in peptidoglycan biosynthesis (63). Recently, it was shown that the *trans-*editing activity of MurM does not require the presence of its second lipid substrate (61) and this protein is able to deacylate not only l-Ala/l-Ser-tRNA^Ala^ but also l-Ala-tRNA^Ser^/tRNA^Phe^/tRNA^Lys^ and Ser-tRNA^Phe^ (64). Thus, MurM is a mediator between the cell wall modification system, translation quality control and a stringent response to environmental stresses (64). The FemABX family of non-ribosomal peptidyltransferases (65) has a unique catalytic mechanism (66). The specificity of FemXs depends mainly upon the sequence of the tRNA, although the wobble base pair G3:U70, the main identity determinant of AlaRS, is not essential for FemX recognition (67). These aminoacyl transferases discriminate between Ala-tRNA^Ala^ and Ser-tRNA^Ser^. Experiments with d-Ala/l-Ala-helixes^Ala^ have demonstrated the catalytic prevalence of substrates with l-Ala (68).

Our study demonstrates that both *T. thermophilus* AlaRS and DTD possess hydrolytic activity against cognate l-Ala-tRNA^Ala^ and noncognate Gly-tRNA^Ala^ (Figure 4). Importantly, the mechanisms of deacylation of mischarged tRNA^Ala^ with d-Ala were not described previously. This study reveals a hitherto unknown function of the AlaRS editing domain in chirality control during translation. The data indicate that AlaRS effectively hydrolyzes the misacylated d-Ala-tRNA^Ala^ substrates (Figure 4).

We hypothesize that together with AlaX-S and DTD, MurM and FemX (66) were the ancestor *trans-*editing factors, controlling the levels of charged tRNA^Ala^ with cognate and noncognate amino acids, therefore providing the proper cellular concentrations of free Ser, Gly and Ala. All these structurally distinct enzymes demonstrate that the main chain atoms of amino acid residues are required for their catalytic function and represent relaxed substrate specificity.

Most likely, in the process of evolution, there was a gradual fixation of homochirality in protein biosynthesis. Two main scenarios of such fixation could be postulated from our data. In the first scenario, DTD appeared to assist such fixation in the early stages of evolution. In the second scenario, which is more likely, several DTD enzymes appeared later and ‘cooperated’ with aaRSs in establishing the chiral selectivity of the translation apparatus. According to the first scenario, all DTDs must have originated from a common predecessor. However, there are several types of DTDs (DTD1, DTD2, DTD3 and ATD), most of which are structured differently (53,69,70). This variation supports the second scenario.

Regardless of the origin of the first amino acid (high electric charge, volcanic or meteoritic origin), Nature encountered a high concentration of small amino acids—glycine and alanine—and their d-enantiomers (d/l-Ala) during the chemical evolution and the early stages of biological evolution (3). So, the first ancestor of AlaRS was required to solve the problem of misacylation of tRNA^Ala^ with both Gly and d-Ala (also with Ser), because the inclusion of precisely these erroneous amino acids drastically changed the structure of the synthesized peptide or protein. Therefore, it is likely that the precursor of AlaRS (or AlaX, and then AlaRS itself) could have acquired editing activity against both Gly and d-Ala.

Thus, it can be assumed that two different strategies for establishing the chiral selectivity of translation apparatus potentially exist: with and without the participation of DTDs, hereupon demonstrating the ‘ancient’ and ‘early’ fidelity control mechanisms. In this line, our findings support the early (Ala) and late (Tyr) scenarios for aaRSs’ evolution, confirming the development of different apparatus for editing factors.

## Supplementary Material

gkz756_Supplemental_File
