# Supplementary material for: Stereospecificity control in aminoacyl-tRNA-synthetases: new evidence of d-amino acids activation and editing
Source: Nucleic Acids Res. 2019 Sep 3;47(18):9777–88. doi: 10.1093/nar/gkz756 (PMC6765224; doi:10.1093/nar/gkz756)
Supplement: gkz756_Supplemental_File [file gkz756_supplemental_file.docx]

Supplementary Information for

**Stereospecificity control in aminoacyl-tRNA-synthetases: new evidence of D-amino acids activation and editing**

Mariia Yu. Rybak*, Alex V. Rayevsky, Olga I. Gudzera and Michael A. Tukalo^*^

Department of Protein Synthesis Enzymology, Institute of Molecular Biology and Genetics of the NAS of Ukraine, 150 Zabolotnogo Str., 03143, Kyiv, Ukraine

Corresponding authors: Michael A. Tukalo and Mariia Yu. Rybak

Emails: [mtukalo@imbg.org.ua](mailto:mtukalo@imbg.org.ua) / [mtukalo1@gmail.com](mailto:mtukalo1@gmail.com); [mariia.rybak@gmail.com](mailto:mariia.rybak@gmail.com)

**This PDF file includes:**

Figs. S1 to S5

Table S1

References for Supplementary Information

**Other supplementary materials for this manuscript include the following:**

Additional data S1 (separate file)

**Figure S1: Confirmation of** d**-Ala and** d**-Ser activation by AlaRS**


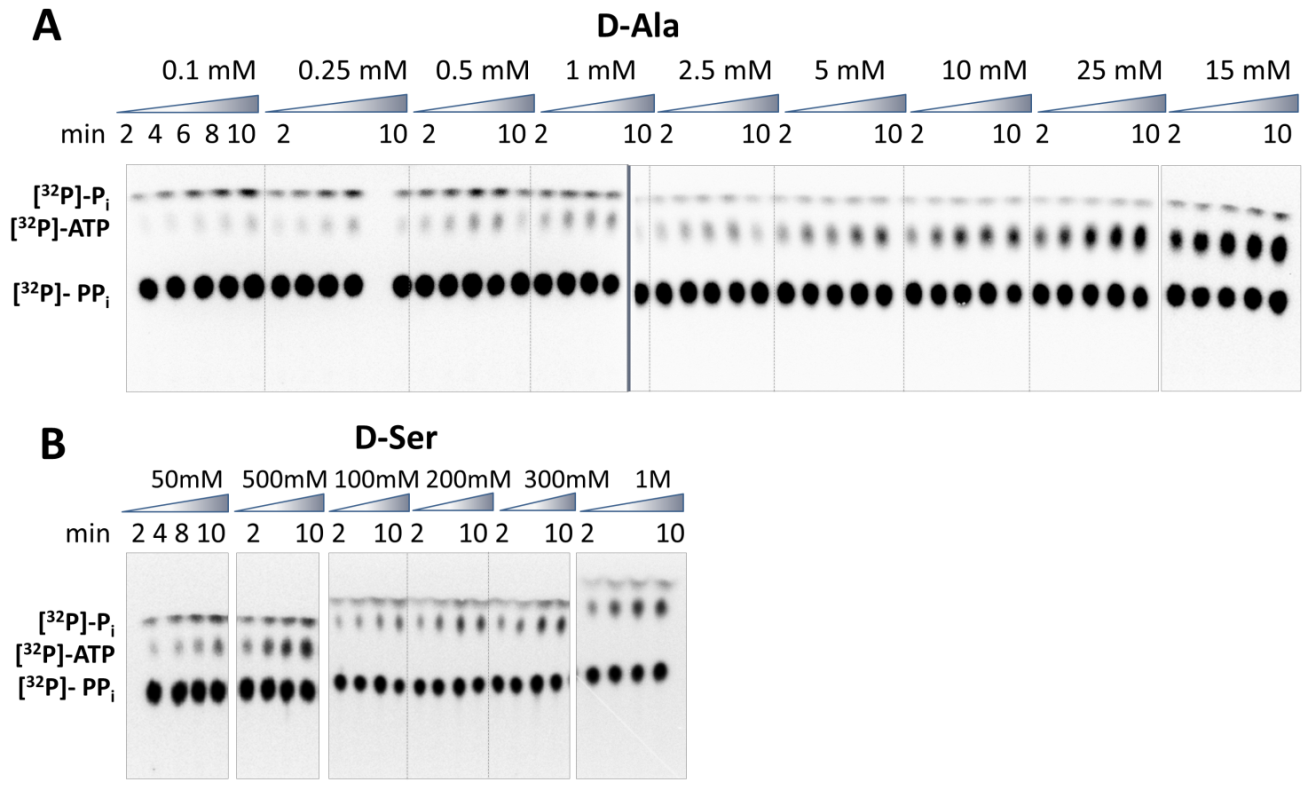


Figure S1: TLC of ATP-PPi assay for d-Ala (1 µM AlaRSTT) and d-Ser (2.5 µM AlaRSTT)

**Figure S2: Comparison of amino acids specificity rates for *E. coli* and *T. thermophilus* AlaRS**


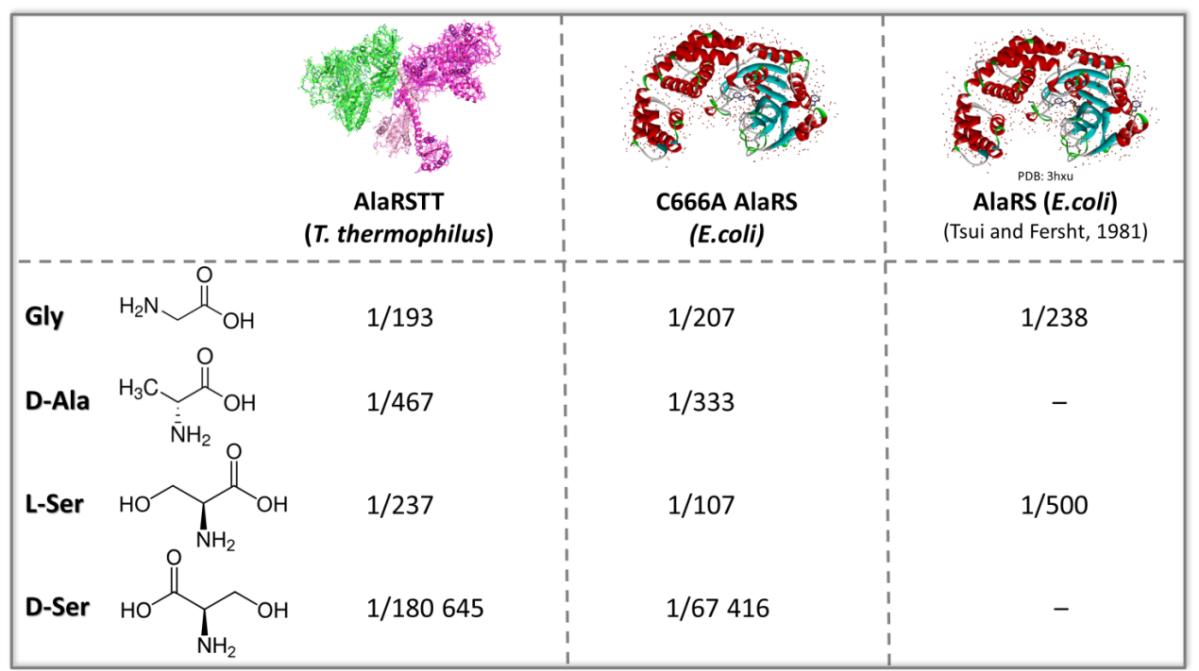


Figure S2. Scheme of catalytic efficiency rates for noncognate amino acids, misactivated by AlaRSTT and *E. coli* C666A AlaRS (current study, ATP-PPi exchange assay) in comparison with literature data (1)

**Figure S3: Interaction energy between the AlaRSTT binding site and l-/d-aminoacyl-adenylates**


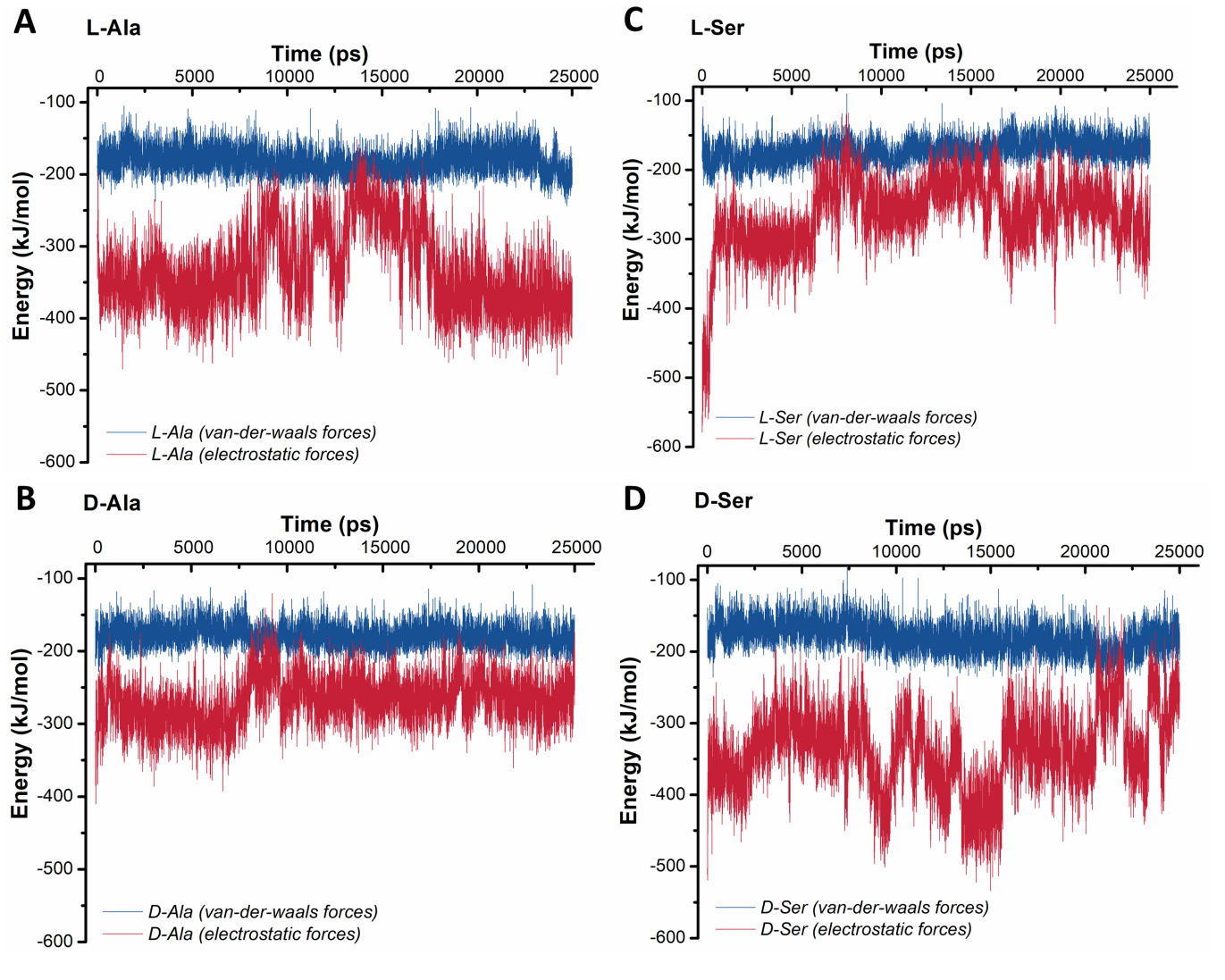


Figure S3. The comparative analysis of interaction energy between the AlaRSTT binding site and ~~4~~ four types of aminoacyl-adenylates. All four substrates demonstrate a similar level of Van-der-Waals interaction energy, defined with its purine moieties, which fluctuates at around –200kJ/mol. However, electrostatic interaction values are different, and those of the l-aminoacyl-adenylates (A, C) probably are more favorable, especially alanyl-adenylate, despite the fluctuation at the midpoint section of the trajectory/graph. Meanwhile, d-stereoisomers (B, D) are in a less beneficial situation with lower values and a leapfrogging oscillation.

**Figure S4: RMSD and RMSF plots reflecting protein (AlaRS) structure with l/d-adenylates**

**
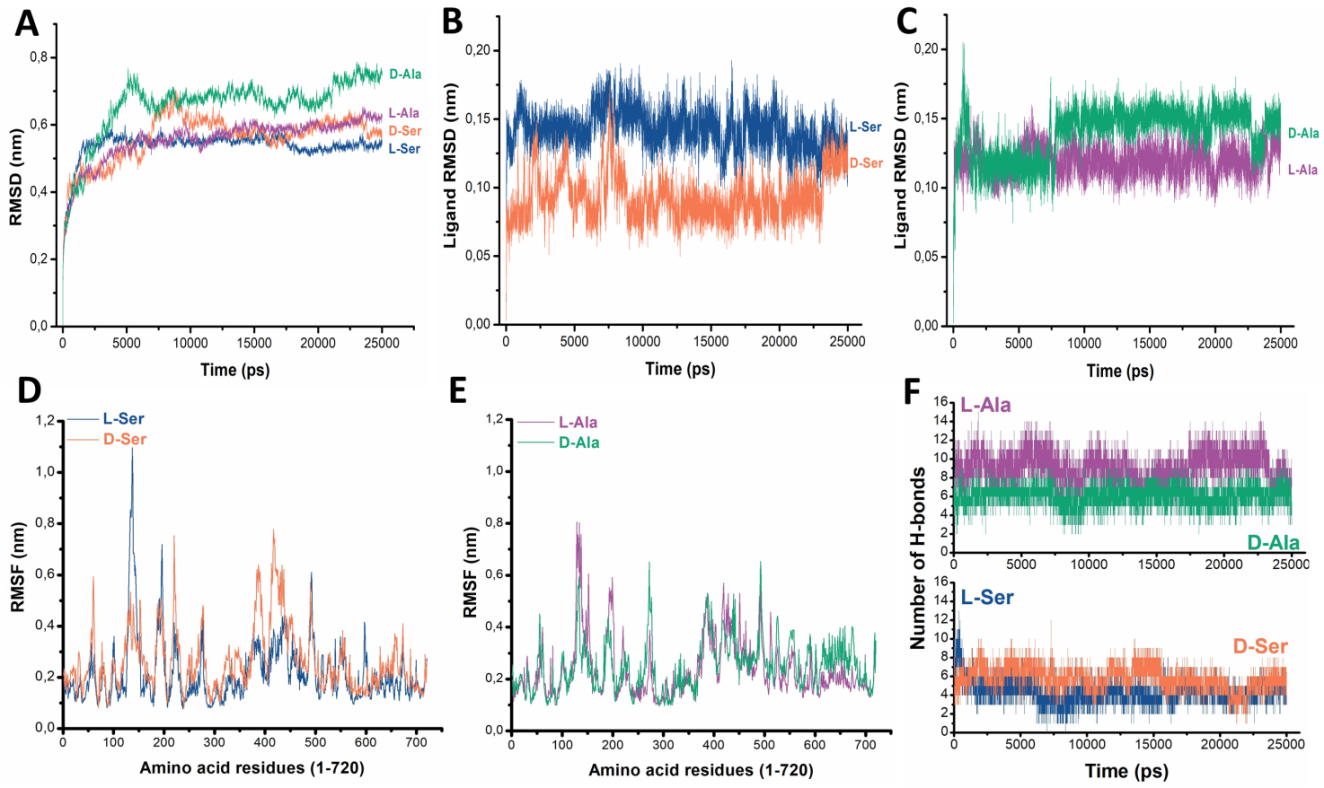
**

Figure S4. The RMSD plot (A) reflects the level of stability for the protein structure with different substrates along with the 25 ns MD simulation. Corresponding RMSD graphs of seryl-adenylates (B) and alanyl-adenylates (C) show significant positional stability of l- compared with d-stereoisomers. The analysis of single residue motions resulted in two root mean square fluctuation (RMSF) plots for seryl-adenylates (D) and alanyl-adenylates (E). From these plots, it can be seen that d-stereoisomers cause dramatic flexibility of the overall structure, while l-forms do not possess such flexibility. It may be that a cognate l-alanyl-adenylate determines even the less pronounced motility of the binding site residues. Hydrogen-bonding scatter (F) demonstrates the same trend in an interaction representation, namely the advantages in the binding of l-stereoisomers over the d-stereoisomers.

**Figure S5: AMP formation over time by WT AlaRS**

**
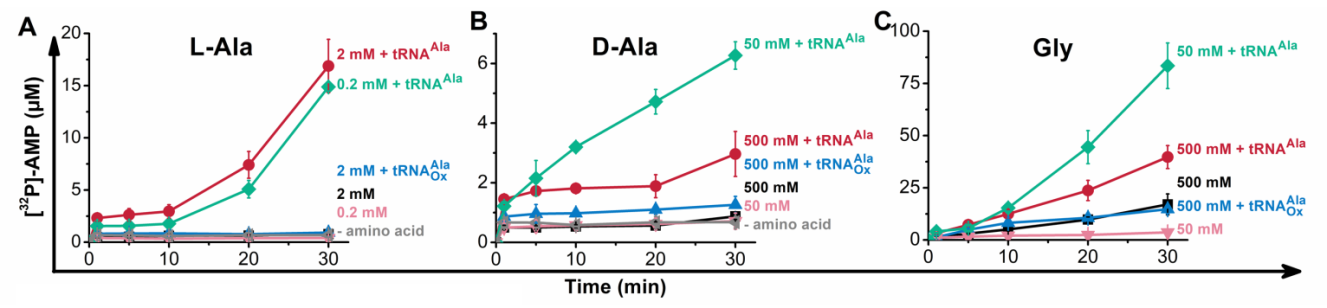
**

Figure S5. Time-course AMP formation by WT AlaRSTT (1 µM) at 37 °C (pH 7.5): for l-Ala (A), d-Ala (B) and Gly (C). tRNA^Ala^_Ox_—oxidized tRNA^Ala^ by NaIO_4_

**Table S1** **Steady-state parameters for amino acid activation by *E. coli* C666A AlaRS**

| Amino acid | *k*_cat_ (s^–1^) | *K*_m_ (mM) | *k*_cat_ / *K*_m_  (mM^–1^ s^–1^) | Discrimination factor |
| --- | --- | --- | --- | --- |
| l-Ala^a^ | 0.18 ± 0.04 | 0.304 ± 0.043 | 0.6 | 1 |
| d-Ala^b^  Gly^c^  l-Ser^d^  d-Ser^e^ | 0.1 ± 0.04  0.47 ± 0.16  0.147 ± 0.04  0.0046 ± 0,00007 | 60 ± 20  182.5 ± 60  27 ± 6  517 ± 160 | 1.8*10^-3^  2.9*10^-3^  5.6*10^-3^  8.9*10^-6^ | 333  207  107  67 416 |
| The data represent mean values ± S.E. (n = 3), measured by ATP-PPi exchange assay.  ^a^ C666A AlaRS was assayed at 250 nM concentration.  ^b^ C666A AlaRS was assayed at 5 µM concentration.  ^c^ C666A AlaRS was assayed at 300 nM concentration.  ^d^ C666A AlaRS was assayed at 2.5 µM concentration.  ^e^ C666A AlaRS was assayed at 10 µM concentration.  [l-Ala] was in the range from 10 µM to 4 mM, [d-Ala] – from 2 mM to 1 M, [Gly] – from 0.5 mM to 1 M, [l-Ser] – from 5 mM to 1M, [d-Ser] – from 50 mM to 1 M.  The concentrations of l-/d-Ala, Gly, l-/d-Ser varied over the range 0.03-13 *K*_m_ (L-Ala), 0.03-17 *K*_m_ (d-Ala), 0.03-6 *K*_m_ (Gly), 0.19-19 *K*_m_ (l-Ser) and 0.1-1.9 *K*_m_ (d-Ser). The highest d-Ser concentration (1.9 *K*_m_, 1M) was limited by its weak solubility.  Discrimination factor = (*k*_cat_ /*K*_m_)l_-Ala (cognate amino acid)_ / (*k*_cat_ /*K*_m_)_noncognate_ | | | | |

Table S1: Steady-state parameters for amino acid activation by *E. coli* C666A AlaRS at 37 °C (pH 7.5) show similar specific specificity rates for d-Ala, Gly, l-/d-Ser comparing to *T. thermophilus* AlaRS.

**References for Supplementary Information**

1. Tsui W-C & Fersht AR (1981) Probing the principles of amino acid selection using the alanyl-tRNA synthetase from *Escherichia coli*. *Nucleic acids research* 9(18):4627-4637.

**Additional data S1 (separate file).**

Framer (script for Python) for water calculations in the DTDTT active site (for Fig.5 D-I)

import numpy as np

# place this scriptfile into the folder with a processible file

__location__ = os.path.dirname(__file__)

inputfile = input('Enter your input file name : ')

outputfile = input('Enter your output file name : ')

ligname = input('Enter your ligand name in *.pdb : ')

carbonname = input('Enter the carbonyl carbon of the ligand : ')

oxygenname = input('Enter the carbonyl oxygen of the ligand : ')

solname = input('Enter the water name: ')

OW = input('What is Your water oxygen atom name? ')

H1 = input('What is Your water hydrogen 1 atom name? ')

H2 = input('What is Your water hydrogen 2 atom name? ')

def remove_doubled(dicty):

to_add = []

for k,v in dicty.items():

if len(v) == 6: # whether the solvent molecule in dict? Does it have three atoms?

# if yes, Rename key with index dash

renamedsol = k + '_'

to_add.append((renamedsol, [v[0], v[2], v[4]])) # sol[renamedsol] = sol.pop(namesol)

to_add.append((k, [v[1], v[3], v[5]]))

for i in to_add:

dicty[i[0]] = i[1]

return dicty

def distance(molecule, atom1):

# atom coordinate x1, y1, z1

x2,y2,z2 = float(atom1[0]), float(atom1[1]), float(atom1[2])

distA1 = 0

distA2 = 0

distA3 = 0

atom2 = []

atom3 = []

a = 0

b = np.array([x2,y2,z2])

for x in molecule :

if OW in x[0]:

# distance from OW to atom

x1,y1,z1 = float(x[1][0]), float(x[1][1]), float(x[1][2])

distA1 = (((x1-x2)**2)+((y1-y2)**2)+((z1-z2)**2))**(1/2)

a = np.array([x1,y1,z1])

if H1 in x[0]:

# print(x)

x1,y1,z1 = float(x[1][0]), float(x[1][1]), float(x[1][2])

distA2 = (((x1-x2)**2)+((y1-y2)**2)+((z1-z2)**2))**(1/2)

atom2 = [x1,y1,z1]

if H2 in x[0]:

x1,y1,z1 = float(x[1][0]), float(x[1][1]), float(x[1][2])

distA3 = (((x1-x2)**2)+((y1-y2)**2)+((z1-z2)**2))**(1/2)

atom3 = [x1,y1,z1]

output = [a, b, distA1, distA2, distA3, atom2, atom3]

if distA1 <= 3.5 and distA1 < distA2 and distA1 < distA3: # distA1 is a changable value

return output

def process(sol, prot, carbon, oxy):

# oxy coordinate x3, y3, z3

x3,y3,z3 = float(oxy[0]), float(oxy[1]), float(oxy[2])

satisfy_req = []

for k,v in sol.items():

first_shell = distance(v, carbon)

if first_shell != None:

# first_shell

# call for function distance and declare vars

a, b, distOW_a, distHW1_a, distHW2_a, HW1, HW2 = \

first_shell[0], first_shell[1], first_shell[2], first_shell[3], first_shell[4], first_shell[5], first_shell[6]

c = np.array([x3,y3,z3])

ba = a - b

bc = c - b

if distOW_a <= 3.5: # does it meet a distance requirement

cosine_angle = np.dot(ba, bc) / (np.linalg.norm(ba) * np.linalg.norm(bc))

angle = np.arccos(cosine_angle) # angle between vectors in degrees

degree = np.degrees(angle)

satisfy_req.append('water_distance')

if degree >= 104 and degree <= 110: # test line was - if degree >= 80 and degree <= 130:

h_first_shell = [HW1, HW2]

satisfy_req.append(k)

for item in h_first_shell: # angle requirement is passed, so secon shell is started

for k,v in sol.items():

second_water = k

second_shell = distance(v, item)

if second_shell != None:

h_second_shell = [second_shell[i] for i in (5, 6)]

if len(h_second_shell) != None:

for n in h_second_shell:

for k,v in prot.items():

for x in v:

# third shell - protein identification

x1,y1,z1 = float(x[0]), float(x[1]), float(x[2])

x2,y2,z2 = float(n[0]), float(n[1]), float(n[2])

distX = (((x1-x2)**2)+((y1-y2)**2)+((z1-z2)**2))**(1/2)

if distX <= 3.5:

envi = k + '_' + second_water

if envi not in satisfy_req:

satisfy_req.append(envi) # assistant molecule and protein

return satisfy_req

frame_analysis = {}

res = ('ARG', 'ASN', 'ASP', 'ASX', 'CYS', 'GLU', 'GLN', 'GLX', 'GLY', 'HIS', 'ILE', 'LEU', 'LYS', 'MET', 'PHE', 'PRO', 'SER', 'THR', 'TRP', 'TYR', 'VAL')

with open(inputfile,'r') as inp:

count = 0

frame = False # a key to scratch the frame and start math operations

sol = {} # water dictionary - 'SOL_152' : [('18.710','15.140','15.270'),..]

prot = {}

carbon = [] # first node vector

oxy = [] # second node vector

for line in inp:

abbr = ' '.join(line.split()) # multispacer ' ' -> ' '

lspl = abbr.split(' ') # ['ATOM','4331','CG1','VAL','134','42.615','59.033','19.339','1.00','0.00']

if 'MODEL' in lspl:

frame = True # determines a new frame to read and needs for 'MODEL' strings

count += 1

elif frame == True:

# frame_slice.append(line)

# starts a division on groups

if solname in lspl:

namesol = '_'.join(lspl[3:5]) # 'SOL' and '151'

if namesol in sol:

atomname = lspl [2] + '_' + lspl [3] + '_' + lspl [4]

atom = (atomname, lspl[5:8]) # atomtype + coordinates

sol[namesol].append(atom) # add next atom to water molecule

else:

atomname = lspl [2] + '_' + lspl [3] + '_' + lspl [4]

atom = (atomname, lspl[5:8]) # ([atomtype], [coordinate])

sol[namesol] = [atom] # molecule ID: [([atomtype], [coordinate]), ]

elif ligname in lspl: # ligand identifier

if carbonname == lspl[2]: # target carbon name

carbon = (lspl[6:9])

elif oxygenname == lspl[2]:

oxy = (lspl[6:9]) # target oxygen name

elif 'ENDMDL' in lspl:

sol = remove_doubled(sol)

frame_analysis[count] = process(sol, prot, carbon, oxy)

frame = False

carbon =[]

sol = {}

prot = {}

oxy = []

elif 'TER' in lspl: pass

else: # protein_amino acid environment identification

for i in res:

if i in lspl:

if 'O' in lspl[2] or 'N' in lspl[2] and not 'H' in lspl[2]:

nameprot = lspl[3] + '_' + lspl[4] + '_' + lspl[5]

if nameprot in prot:

prot[nameprot].append((lspl[6:9]))

else:

prot[nameprot]= [(lspl[6:9])]

with open(outputfile,'w') as out: # generation of the output list

for k,v in frame_analysis.items():

frame = str(k) + '\t'

incl = '\t'.join(v)

out.write(frame + incl + '\n')
